# Administration of Bone Marrow-Derived Mononuclear Cells Contributed to the Reduction of Hypoxic-Ischemic Brain Injury in Neonatal Rats

**DOI:** 10.3389/fneur.2018.00987

**Published:** 2018-11-30

**Authors:** Yoshiaki Sato, Kazuto Ueda, Taiki Kondo, Tetsuo Hattori, Alkisti Mikrogeorgiou, Yuichiro Sugiyama, Toshihiko Suzuki, Michiro Yamamoto, Hitoshi Hirata, Akihiro Hirakawa, Keiko Nakanishi, Masahiro Tsuji, Masahiro Hayakawa

**Affiliations:** ^1^Division of Neonatology, Center for Maternal-Neonatal Care, Nagoya University Hospital, Nagoya, Japan; ^2^Department of Hand Surgery, Nagoya University Graduate School of Medicine, Nagoya, Japan; ^3^Department of Biostatistics and Bioinformatics, Graduate School of Medicine, The University of Tokyo, Tokyo, Japan; ^4^Department of Perinatology, Aichi Human Service Center, Institute for Developmental Research, Aichi, Japan; ^5^Department of Regenerative Medicine and Tissue Engineering, National Cerebral and Cardiovascular Center, Osaka, Japan

**Keywords:** infant, encephalopathy, cell therapy, regenerative medicine, cerebral palsy, mental retardation

## Abstract

**Background/Objective:** Perinatal hypoxic-ischemia (HI) causes neonatal death and permanent neurological deficits. Cell therapy using various cell sources has been recently identified as a novel therapy for perinatal HI. Among the available types of cell sources, bone marrow-derived mononuclear cells (BMMNCs) have unique features for clinical application. For example, stem cells can be collected after admission, thus enabling us to perform autologous transplantation. This study aimed to investigate whether the administration of BMMNCs ameliorated HI brain injury in a neonatal rat model.

**Methods:** Seven-day-old rats underwent left carotid artery ligation and were exposed to 8% oxygen for 60 min. BMMNCs were collected from the femurs and tibias of juvenile rats using the Ficoll–Hypaque technique and injected intravenously 24 h after the insult (1 × 10^5^ cells). Active caspase-3, as an apoptosis marker, and ED1, as an activated microglia/macrophage marker, were evaluated immunohistochemically 48 h after the insult (vehicle, *n* = 9; BMMNC, *n* = 10). Behavioral assessments using the rotarod treadmill, gait analysis, and active avoidance tests were initiated 3 weeks after the insult (sham, *n* = 9, vehicle, *n* = 8; BMMNC, *n* = 8). After these behavioral tests (6 weeks after the insult), we evaluated the volumes of their hippocampi, cortices, thalami, striata, and globus pallidus.

**Results:** The mean cell densities of the sum of four parts that were positive for active caspase-3 significantly decreased in the BMMNC group (*p* < 0.05), whereas in the hippocampi, cortices, thalami, and striata cell densities decreased by 42, 60, 56, and 47%, respectively, although statistical significance was not attained. The number of ED1 positive cells for the sum of the four parts also significantly decreased in the BMMNC group compared to the vehicle group (*p* < 0.05), whereas in each of the four parts the decrease was 35, 39, 47, and 36%, respectively, although statistical significance was not attained. In gait analysis, the BMMNC normalized the contact area of the affected hind paw widened by HI. The volumes of the affected striata and globus pallidus were significantly larger in the BMMNC group than in the control group.

**Conclusion:** These results indicated that the injection of BMMNCs ameliorated HI brain injury in a neonatal rat model.

## Introduction

Neonatal encephalopathy (NE) is a neurological syndrome that presents with clinical features consistent with that of brain disorders. Its most relevant clinical features are decreased consciousness and respiratory depression, abnormal strength and muscle tone, impaired feeding, and seizures (1). In developed countries, the incidence of NE is ~1–6 per 1,000 live births (2). NE is frequently associated with acute hypoxic-ischemic insults, and 50–80% of cases are considered to have hypoxic-ischemic encephalopathy (HIE) (1). Despite the developments in perinatal medicine, perinatal asphyxia remains an important cause of neonatal death and permanent neurological deficits (3, 4), which currently has no effective treatments other than hypothermia. Previous randomized trials have shown that hypothermia reduces the risk of death or disability in infants with HIE (5). However, it has not been found effective in cases of severe HIE (6).

Stem cell therapy is expected to have applications in the treatment of central nervous system diseases (7), and several types of stem cells constitute potential sources of cell therapy for future clinical applications. We have previously demonstrated that intracerebroventricular injection consisting of neural stem/progenitor cells (NSPCs) with chondroitinase ABC, which digests glycosaminoglycan chains on chondroitin sulfate proteoglycans, reduces brain injury in a rat model of neonatal hypoxic-ischemia (HI) (8, 9). Additionally, Ji et al. (10) reported the effects of intranasal treatment with engrafted NSPCs. However, ethical and safety concerns hinder the use of NSPCs derived from the brain of a human fetus in clinical practice (11). Ethical considerations can be avoided through the use of stem cells originating from non-neural tissues such as umbilical cord blood cells (UCBCs), which are readily available and can be exploited for autologous transplantations. We have recently confirmed the beneficial effects of UCBC-derived mononuclear cells in a rat model of neonatal HI and in a mouse model of stroke (12–14). The subsequent clinical trials are currently under way (ClinicalTrials.gov: NCT02256618). Although autologous UCBCs are a promising source for stem cell therapy against NE, their collection may be difficult during emergencies, such as precipitous delivery.

Bone marrow contains populations of multipotent precursors, including mesenchymal cells, that can differentiate into multiple cell types (15). The ability of bone marrow cells to differentiate into neurons and glia was recently demonstrated (16), as was their ability to cross the blood brain barrier and preferentially enter the brain upon intravenous infusion (17). Bone marrow-derived mononuclear cells (BMMNCs) have been reported to reduce neurological impairments in a rat model of adult ischemic stroke (18). BMMNCs constitute a promising source for cellular therapy, since they can be rapidly collected and isolated from bone marrow after admission. Moreover, they are enriched with stem cells, allowing autologous application, and the feasibility and safety of their harvest and reinfusion has been previously demonstrated by a clinical trial in acute stroke patients (19). However, to our knowledge, no studies have studied the effect of BMMNCs in a rat model of neonatal HI. The present study investigated the effects of BMMNCs administration to neonatal HI rats.

## Materials and methods

### Animals

All animal experimental protocols in the present study were approved by the Institutional Review Board of Nagoya University School of Medicine (Nagoya, Aichi Prefecture, Japan; permit No.: 23181, 24337, 25170, 26128). Only male Wistar/ST rat pups were acquired from Japan SLC Inc. (Shizuoka, Japan) to avoid the possible behavioral effects of the sexual cycle in female rats (20–22). The rats were housed under a 12-h light/dark cycle (lights were kept on from 8:00 a.m. to 8:00 p.m.) with *ad libitum* access to food and water. The animal room and experimental space were maintained at 23°C.

### BMMNC preparation

For each experiment, bone marrow cells were collected from the femurs and tibias of one juvenile Wistar female rat at postnatal day 30 (P30). The bone ends were cut, and the marrow was flushed out using 5 ml of saline heparin solution (200 U heparin/100 ml saline) with a 22 or 23-gauge needle. After washing with 10 ml saline supplemented with 0.1 mM EDTA, the cells were suspended with DMEM/F-12 (Thermo Fisher Scientific, Waltham, Massachusetts, USA) with 2% albumin. Mononuclear cells were isolated using the Ficoll–Hypaque technique (Ficoll-Paque PLUS; GE Healthcare Bio-Sciences AB, Björkgatan, Uppsala, Sweden) and suspended in DMEM/F-12 and 1% fetal bovine serum (FBS) at a concentration of 1 × 10^6^ cells/ml. The cells were administered immediately after collection. Three batches of cells were used, since the experiments were performed on three different dates: two for acute injury evaluation and one for behavioral tests.

### HI insult and BMMNC administration

Hypoxic-ischemic brain damage was induced on P7 rats according to the method of Rice et al. (23) with minor modifications as described previously (13). Briefly, each pup was anesthetized with isoflurane inhalation (5% for induction and 1–2% for maintenance). The left carotid artery was doubly ligated and incised between the ligatures. After a 1-h rest with a dam, the pups were exposed to a hypoxic environment (8% O_2_ and 92% N_2_, at 37°C for 60 min), after which they were returned to the dam in an animal room maintained at 23°C. The pups in the treatment group (BMMNC group; *n* = 10 for acute injury evaluation and *n* = 10 for behavioral tests) were injected with BMMNCs (1 × 10^5^ cells/0.1 ml) intravenously via the right external jugular vein under isoflurane inhalation anesthesia 24 h after the insult. A vehicle group (*n* = 10 for acute injury evaluation and *n* = 10 for behavioral tests) underwent ligation of the left carotid artery and hypoxia in the same manner and received an equivalent volume of DMEM/F-12 and 1% FBS. The sham group (*n* = 3 for acute injury evaluation and *n* = 9 for behavioral tests) underwent neither left carotid artery ligation nor hypoxia. In order to avoid hypothermia, the pups were kept on a water-bath (set to 37°C) before and after the surgery and on a hot plate (set to 37°C) during the surgical procedure.

### Histological and immunohistochemical procedures

Histological and immunohistochemical procedures were performed as previously described (24) with minor modifications. Briefly, rats were deeply anesthetized and intracardially perfusion-fixed with 0.9% NaCl followed by 4% paraformaldehyde in phosphate-buffered saline (PBS) 48 h after the insult. The brains were excised and immersion-fixed in the same solution at 4°C overnight, after which they were dehydrated with a graded series of ethanol and xylene, embedded in paraffin, and cut into 5-μm-thick coronal sections. We then performed antigen retrieval by heating the sections for 10 min in 10-mM citrate buffer (pH 6.0) after deparaffinization and rehydration. Non-specific binding was blocked using 4% donkey serum in PBS. Then, sections were incubated overnight at 4°C with rabbit anti-active caspase-3 (product number 559565; dilution, 1:200; BD Pharmingen, Franklin Lakes, NJ, USA), mouse anti-ED1 (product number MAB1435; dilution 1:300; Merck Millipore, Darmstadt, Germany), or anti-MAP2 (product number MAB3418; dilution 1:400; Merck Millipore) in PBS with 0.1% Triton. Subsequently, the sections were incubated with sufficient biotinylated secondary antibodies (Vector Laboratories, Burlingame, CA, USA) for 1 h at room temperature. Endogenous peroxidase activity was blocked with 3% H_2_O_2_ in PBS for 10 min and then with avidin-biotin-peroxidase complex (Vectastain ABC Elite kit; Vector Laboratories). Peroxidase detection was then performed for 10 min (0.12 mg/mL 3,3-diaminobenzidine, 0.01% H_2_O_2_, and 0.04% NiCl_2_).

### Cell counting for acute injury biomarkers

**Figure 1:**
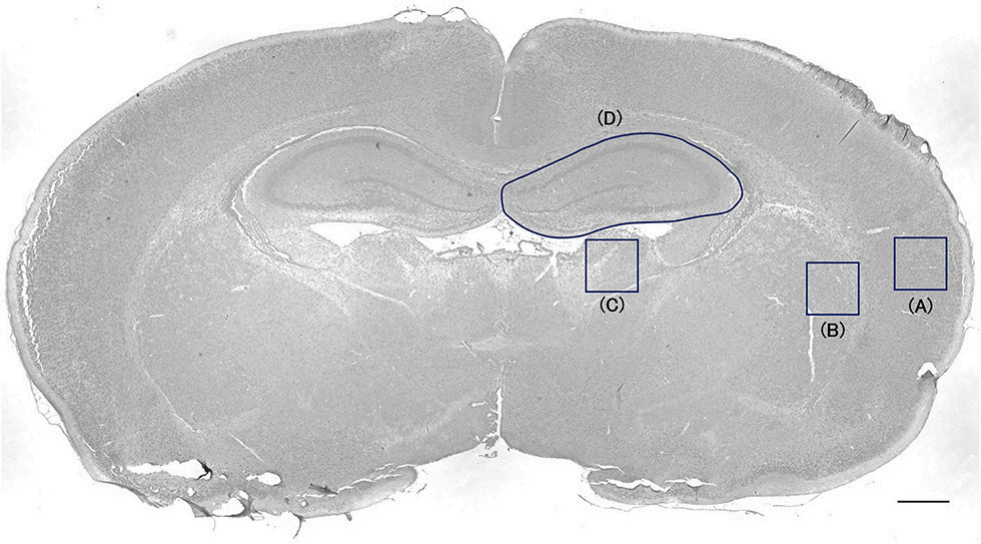
Cell-counting area in the cortex, striatum-pallidum, thalamus, and hippocampus. A square (500 × 500 μm) was placed on the cortex **(A)**, the striatum-pallidum **(B)**, and the thalamus **(C)**. The hippocampus was outlined **(D)**. Bar = 500 μm.

Cells positive for active caspase-3 and ED1 were counted in every 50th section throughout the cortex, striatum-pallidum, thalamus, and hippocampus, resulting in a total of four sections per animal, using the Stereo Investigator version 10 stereology software (MicroBrightField Europe EK, Magdeburg, Germany). A square (500 × 500 μm) was placed on the cortex, the striatum-pallidum, and thalamus, and the hippocampus was outlined (Figure 1). The area was measured in each section. The cortical ROI was placed in the lateral cortex to avoid areas of infarction. The positive cells were counted under high magnification ( × 400) inside each square and outlined hippocampus. Cell counts were expressed as densities. The evaluation was blinded regarding group allocation.

### Behavioral tests

All behavioral tests and evaluations were blinded regarding group allocation.

### Rotarod treadmill

A rotarod treadmill was used to evaluate the motor performance and coordination at P24 and P25. Each rat was placed on a rotating rod (Med Associates Inc., St. Albans, VT, USA), accelerating at 4–40 rpm over 5 min, and the time at which the rat fell from the rod was recorded (maximum 300 s). For 2 consecutive days, the tests were performed twice a day with a 2-h interval.

### Gait analysis

Gait assessment was performed at P31 using the CatWalk quantitative gait analysis system (Noldus Information Technology, Wageningen, The Netherlands) as previously described (13). The experimental rats ran across a glass walkway transversely, and the runs were recorded by a camera positioned below. If an animal failed to complete a run within 5 s, walked backward, or reared during the run, the process was repeated with each rat, and the average of three runs was calculated. The glass walkway was illuminated with beams of light in the dark atmosphere as the animals' paws could reflect light as they touched the glass floor. To calculate the paw-related parameters, each paw was labeled on the recorded video. In the experiment, the contact area (area of paw print) and maximal intensity (the maximal intensity of each paw in the run) were measured.

### Active avoidance test

The active avoidance test was performed from P35 to P38, following the method described by Ichinohashi et al. (25) using the same equipment. Each rat underwent 20 daily sessions of a shuttle avoidance test for 4 consecutive days. The test was conducted in an automated shuttle box (Med Associates Inc., St. Albans, VT, USA), which was divided into two compartments with independently electrified stainless steel bars as a floor. Each session consisted of presenting a buzzer tone and light stimulation (conditioning stimulus, CS) and an electric shock (unconditioned stimulus, US). Both the CS and the US were presented for 5 s, and the US consisted of a positive half-wave constant current of 0.5 mA. Upon presentation of the CS, the rat could avoid the US by escaping to the other compartment of the shuttle box, thus switching off the CS. The interval between each trial varied from 10 to 90 s (30 s on average). The parameters were analyzed using the MED-PC IV software (Med Associates Inc.,). The avoidance proportion, i.e., the number of sessions in which the rat successfully switched off the alert and avoided electric shock, was evaluated each day.

### Volume measurement

After the behavioral tests, rats were deeply anesthetized, intracardially perfusion-fixed, and had their brains excised at P43 using the same histological procedures. The remaining volumes of the hippocampus, thalamus, cortex, striatum, and globus pallidus, were evaluated by staining every 100th section from the whole cerebrum (7–9 sections) with anti-MAP2 antibody. The volumes of each section were calculated according to the Cavalieri principle (Stereo Investigator Ver.10) using the following formula: V = ∑A × P × T, where V = the total volume, ∑A = the sum of area measurements, P = the inverse of the sampling fraction, and T = the section thickness. Section areas with missing regions were regarded as zero. The evaluation was blinded regarding group allocation.

### Statistical analysis

All continuous data were presented as mean ± standard error. The mean body weight gain and acute injury biomarker data in each region were compared between two groups (vehicle vs. BMMNC) using Student's *t*-test. The survival rate, body weight gain, behavioral data, and volume measurement for each region were compared among three groups (sham, vehicle, and BMMNC) using the Dunnett's test. In addition, a two-way ANOVA with the three experimental groups (sham, vehicle, and BMMNC) and four regions as the two independent variables was used to analyze the data pertaining to acute injury biomarker and volume measurement for the sum of the four regions. For acute injury biomarkers, only the vehicle and BMMNC groups were compared since the data of the sham group was prepared only for reference. A *p*-value < 0.05 was considered statistically significant. All statistical analyses were performed using IBM SPSS Statistics 24.0.

## Results

### Safety assessments

At 48 h post-insult 49 rats survived. Three rats died at P7, immediately after hypoxia and at P8 probably due to deep anesthesia. Twenty-two rats were sacrificed at P9 for immunohistological evaluation and 27 rats were maintained for behavioral evaluations. No significant differences in the survival rates were observed among the three groups at P18: 100% (9/9) survived in the sham group, 89% (8/9) in vehicle group, and 100% (9/9) in the BMMNC group. At 6 weeks the survival rates were 100% (9/9) in the sham group, 89% (8/9) in vehicle group, and 89% (8/9) in the BMMNC group. In the vehicle and BMMNC groups one rat died at P17 and one at P21, respectively, which was probably due to HI-derived debilitation.

**Figure 2:**
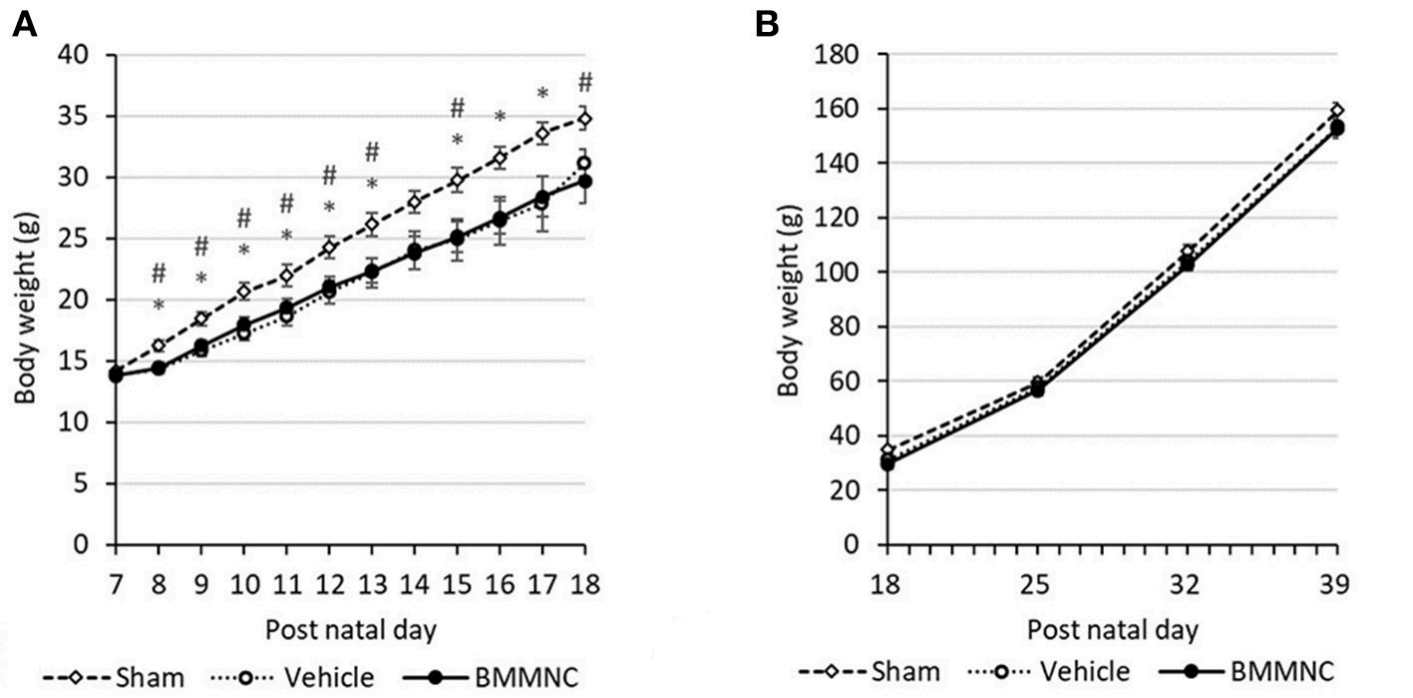
Average weight gain during the acute **(A)** and chronic **(B)** phases. From P8 to P13, the BMMNC and vehicle groups differed significantly from the sham group. No significant difference was observed between the BMMNC and vehicle groups. Open squares and dashed line: sham, *n* = 9; open circles and dotted line: vehicle, *n* = 9 (P7–P17) and 8 (P18–P39); closed circles and solid line: BMMNC, *n* = 9 (P7–P20) and 8 (P21–P39). *Sham vs. Vehicle, *p* < 0.05; # Sham vs. BMMNC, *p* < 0.05; Student's *t*-test.

From P8 to P13, rats that received the HI insult (i.e., those in the vehicle and BMMNC groups), had a lower body weight gain compared to those in the sham group. After injection, the body weight gain did not differ between the BMMNC and vehicle groups (Figures 2A,B). From P25 no differences were observed among the three groups regarding the body weight gain (Figure 2B).

### Impact of BMMNC on the expression of acute injury biomarkers after HI

Twenty-four hours after BMMNCs administration (i.e., 48 h after the insult), active caspase-3 and ED1 were evaluated immunohistologically as an apoptosis and an activated microglia/macrophage marker, respectively, in the hippocampi, cortices, thalami, and striata (Figure 1). Brains without HI injury (*n* = 3) had very few/none positive cells in both markers: active caspase-3; 314 ± 15 cells/mm^3^ in the hippocampus, 67 ± 67 cells/mm^3^ in the cortex, 67 ± 67 cells/mm^3^ in the thalamus, and 133 ± 67 cells/mm^3^ in the striatum-pallidum, ED1; 2,349 ± 316 cells/mm^3^ in the hippocampus, 0 ± 0 cells/mm^3^ in the cortex, 867 ± 133 cells/mm^3^ in the thalamus, and 1,133 ± 742 cells/mm^3^ in the striatum-pallidum.

**Figure 3:**
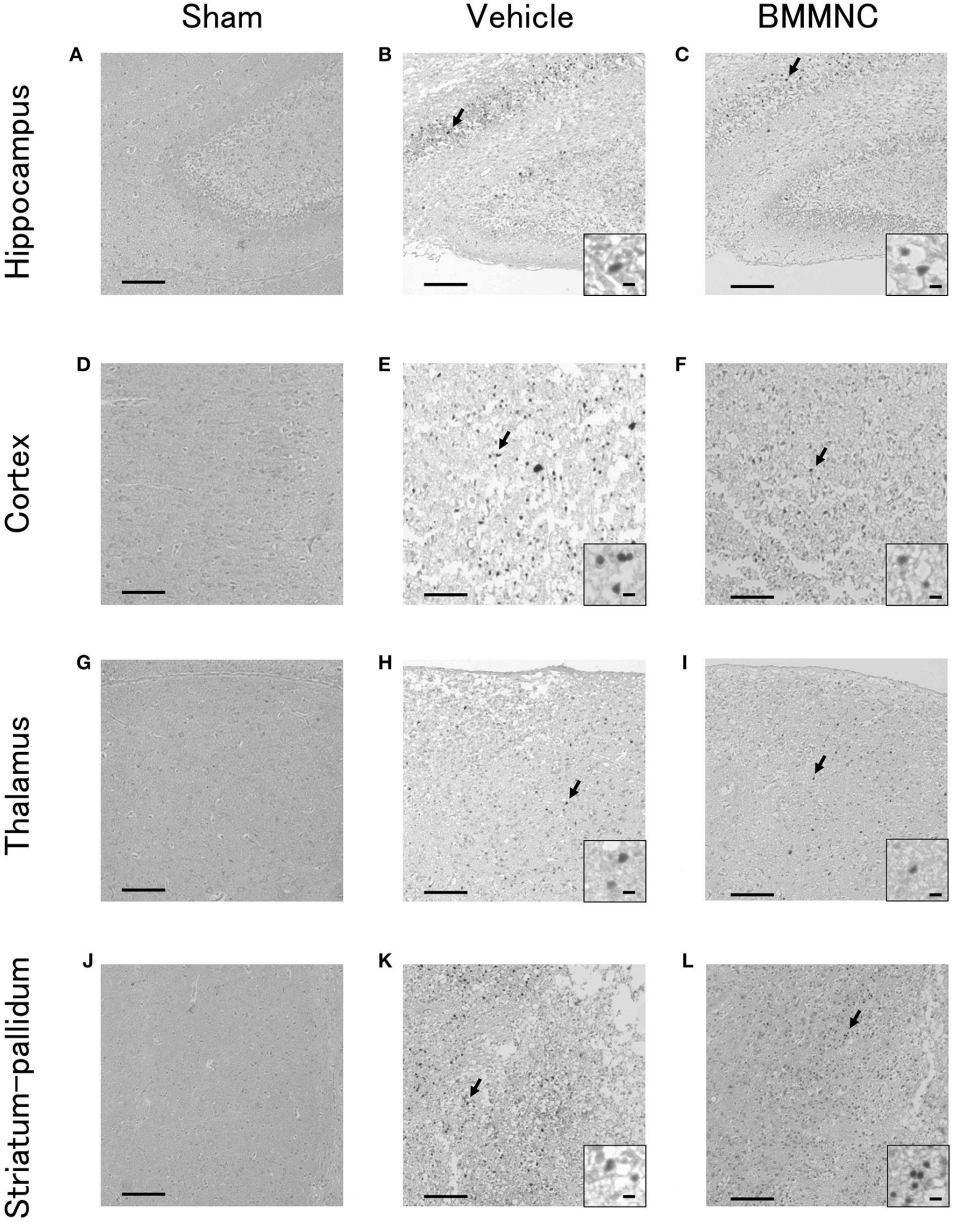
Representative photomicrographs of active caspase-3-positive cells in the hippocampus **(A–C)**, cortex **(D–F)**, thalamus **(G–I)**, and striatum-pallidum **(J–L)** 48 h after HI. Bar = 100 μm. Insets show higher magnification views ( × 400, bar = 5μm).

**Figure 4:**
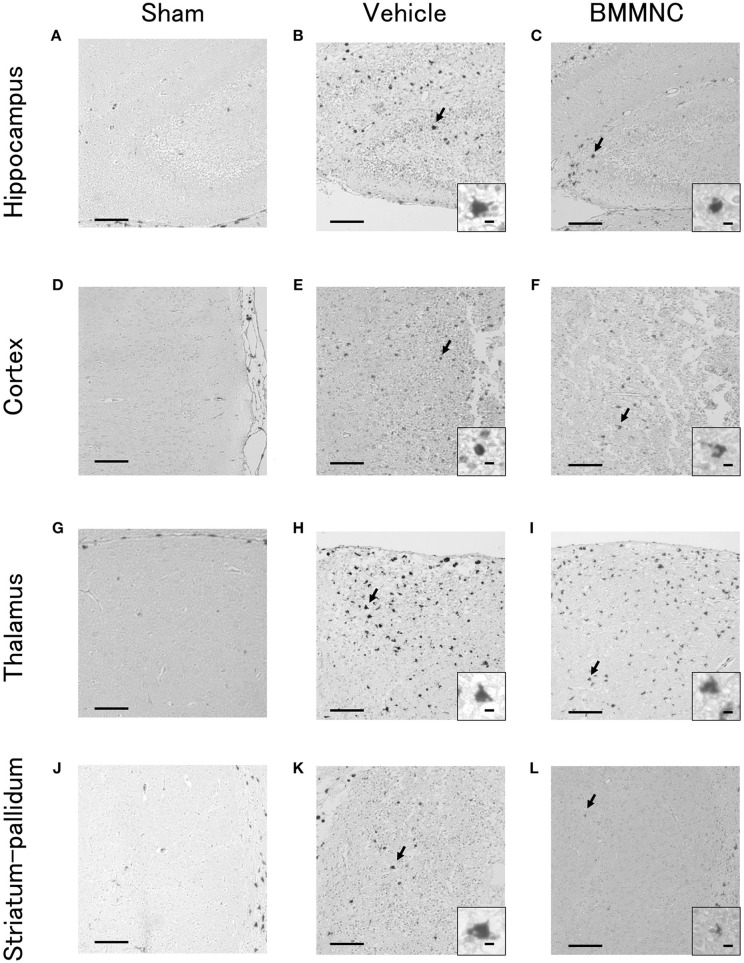
Representative photomicrographs of ED1-positive cells in the hippocampus **(A–C)**, cortex **(D–F)**, thalamus **(G–I)**, and striatum-pallidum **(J–L)** 48 h after HI. Bar = 100 μm. Insets show higher magnification views ( × 400, bar = 5 μm).

Representative photomicrographs of active caspase-3-positive cells in the hippocampus (Figures 3A–C), cortex (Figures 3D–F), thalamus (Figures 3G–I), and striatum-pallidum (Figures 3J–L) are shown, as well as representative photomicrographs of ED1-positive cells in the hippocampus (Figures 4A–C), cortex (Figures 4D–F), thalamus (Figures 4G–I), and striatum-pallidum (Figures 4J–L) are shown.

**Figure 5:**
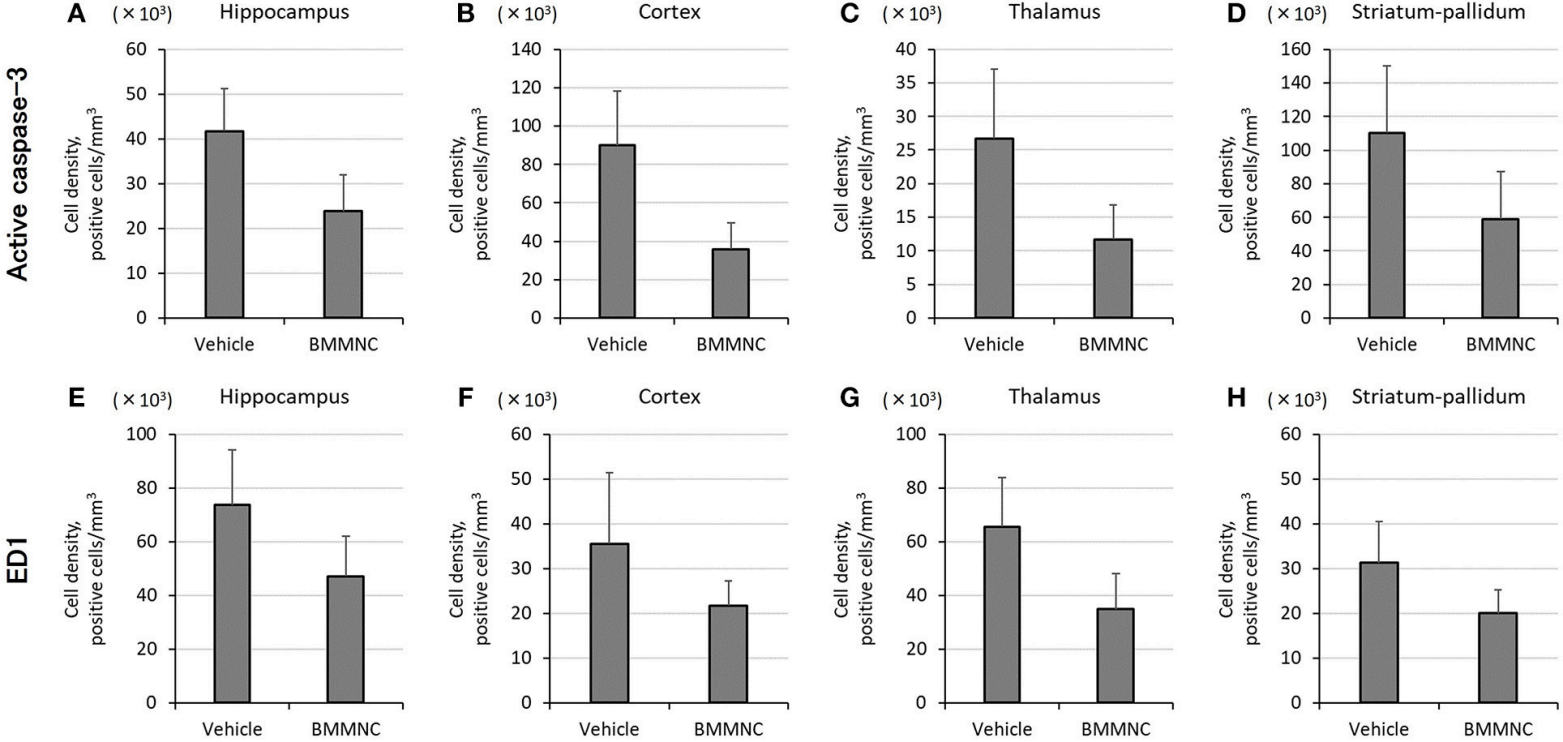
Effect of BMMNC on the expression of acute injury biomarkers for apoptosis [active caspase-3, **(A–D)**] and activated microglia/macrophage [ED1, **(E–H)**]. The number of marker-positive cells was counted in the hippocampus, cortex, thalamus, and striatum-pallidum. The number of active caspase-3-positive cells for the sum of the four parts was significantly lower in the BMMNC group (*n* = 10) than in the vehicle group [*n* = 9, **(A–D)**, two-way ANOVA]. The number of ED1-positive cells for the sum of the four parts was also significantly lower in the BMMNC group [**(E–H)**, two-way ANOVA]. Data are presented as mean ± standard error of the mean.

In the BMMNC group, a significant decrease was observed in the mean cell density for the sum of the four parts that were positive for active caspase-3 (*p* < 0.05, two-way ANOVA model including the two main effects of treatments and regions). On the other hand, in the hippocampi, cortices, thalami, and striata the mean cell density decreased by 42, 60, 56, and 47%, respectively, although the difference did not attain statistical significance (Figures 5A–D).

The number of ED1 positive cells for the sum of the four parts also decreased significantly in the BMMNC group compared with the vehicle group (*p* < 0.05, two-way ANOVA model including the two main effects of treatments and regions). The number of ED1 positive cells in the hippocampi, cortices, thalami, and striata decreased by 35, 39, 47, and 36%, respectively, in the BMMNC group, although the difference did not attain statistical significance (Figures 5E–H).

### Impact of BMMNC on behavior after HI

Two rats were excluded due to HI-derived debilitation and death.

#### Rotarod treadmill

**Figure 6:**
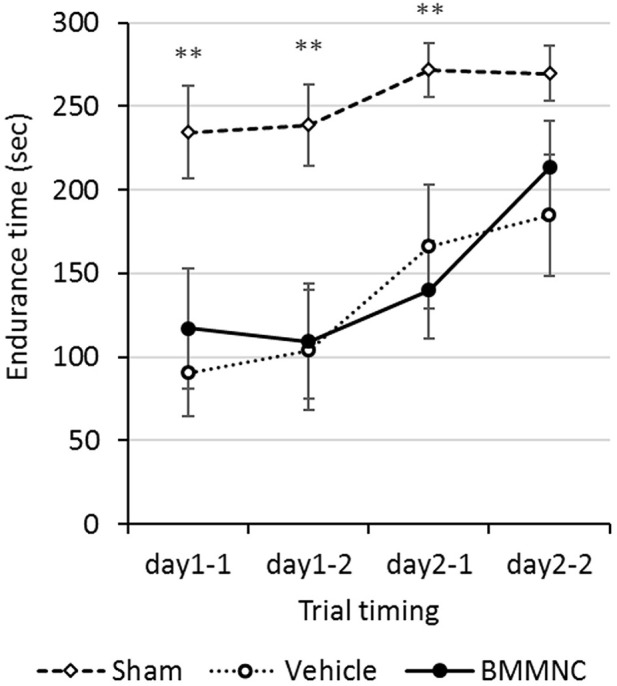
Rotarod treadmill. Rotarod treadmill test was performed on P24–25. The endurance time was compared among all three groups. Rats in the BMMNC group and vehicle group fell down significantly earlier than in those in the sham group from the first to the third trial (**p < 0.01, Dunnett's test). The endurance time was not significantly different between the BMMNC and vehicle groups in any trials. Open squares and dashed line: sham, *n* = 9; open circles and dotted line: vehicle, *n* = 8; closed circles and solid line: BMMNC, *n* = 8.

Motor coordination and motor learning were measured using a rotarod treadmill, 17–18 days after the insult (P24–25). The endurance times were shorter in the BMMNC and vehicle groups than those in the sham group in the first three trials. In the fourth trial (the second trial on day 2), no significant differences were observed among the three groups. Overall, no significant differences were observed between the BMMNC and vehicle groups during all trials (Figure 6).

#### Gait analysis

**Figure 7:**
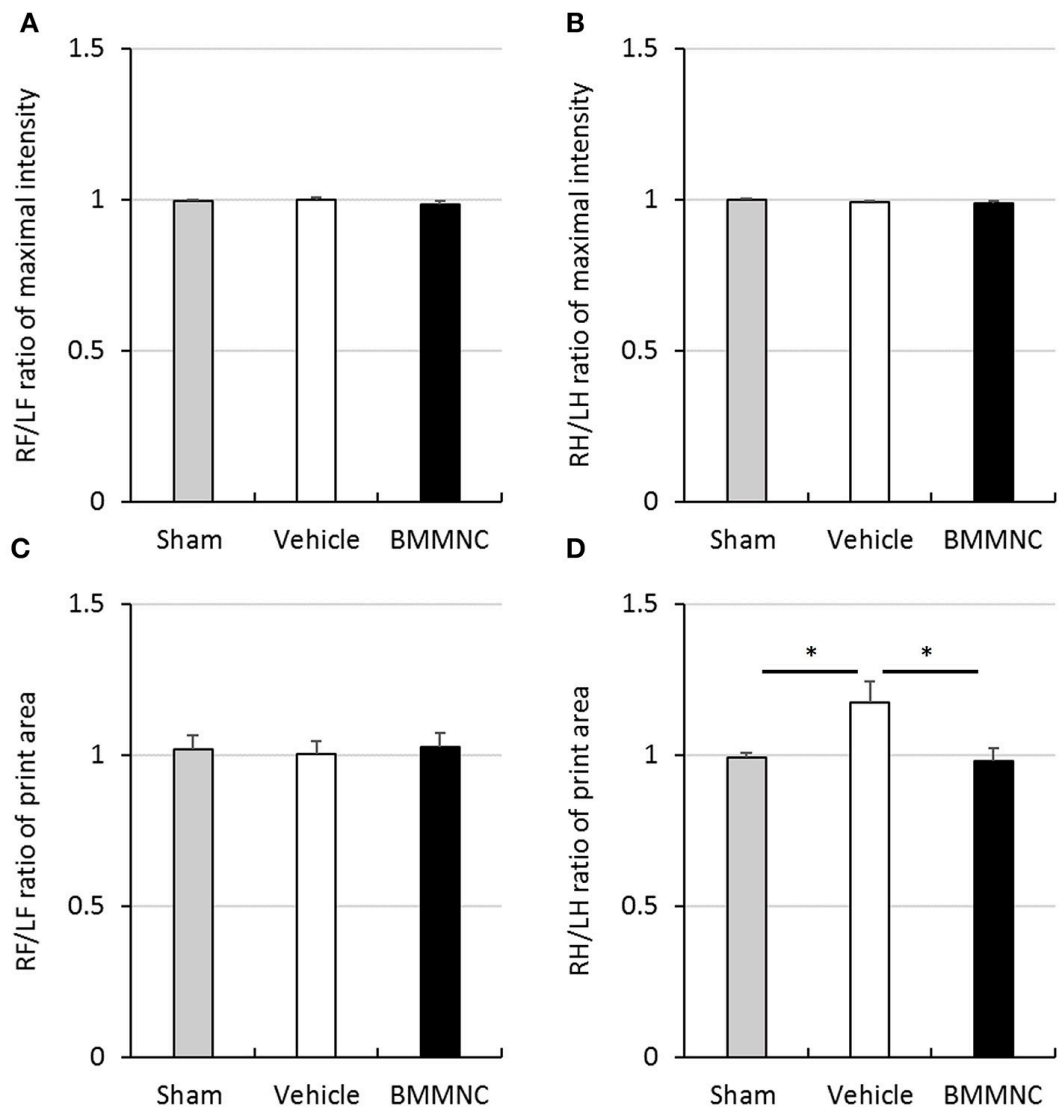
Gait analysis. Gait analysis was performed to assess right/left ratio of the maximal intensity of forepaw **(A)** and hind paw **(B)** and right/left ratio of print area with forepaw **(C)** and hind paw **(D)** on P31. **(A,B)** There was no significant difference in the maximal intensity of forepaw and hind paw. **(C)** A significant difference was not observed in the right/left ratio of print area with forepaw. **(D)** Right/left ratio of print area with hind paw in the vehicle group was significantly larger than that in the sham group (*p < 0.05, Dunnett's test). Moreover, it was significantly smaller in BMMNC group than in the vehicle group (*p < 0.05, Dunnett's test). Data represent the mean ± standard error of the mean (sham, *n* = 9; vehicle, *n* = 8; BMMNC, *n* = 8). RF, right forepaw; LF, left forepaw; RH, right hind paw; LH, left hind paw.

Motor deficits were evaluated 24 days after the insult (P31) through gait analysis using the CatWalk system. No significant differences were observed among the three groups regarding the maximal intensity of each running paw in either hind or fore paws (Figures 7A–B), or the contact areas of the fore paws (Figure 7C). In contrast, the contact areas of the hind paws were significantly larger in the vehicle group than in the sham group, but BMMNC normalized the contact area of the affected hind paw widened by HI (Figure 7D).

#### Active avoidance test

**Figure 8:**
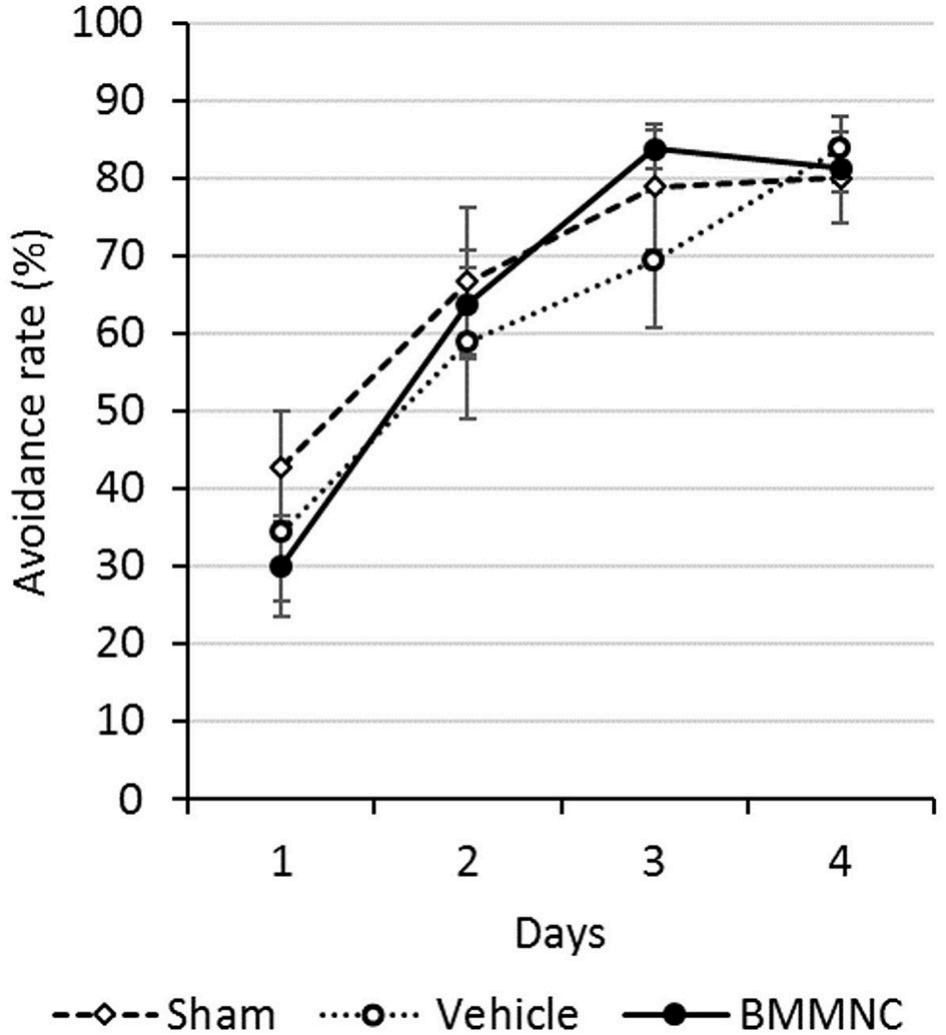
Active avoidance test. The active avoidance test was serially performed on P35–38. The avoidance rates increased with time in all groups. There were no significant differences among all three groups on any day (Dunnett's test). Open squares and dashed line: sham, *n* = 9; open circles and dotted line: vehicle, *n* = 8; closed circles and solid line: BMMNC, *n* = 8. Data represent the mean ± standard error of the mean.

An active avoidance test was performed 28–31 days after the insult (P35–38). The mean avoidance proportion of each group was calculated for 4 consecutive days. The avoidance rates increased with time in all groups, although there were no significant differences among the three groups throughout this period (Figure 8).

### Impact of BMMNC on brain volume after HI

The volumetric assessment was performed for the hippocampus, thalamus, cortex, striatum, and globus pallidus to assess the absolute tissue loss after HI. Sections throughout the whole cerebrum at P43 were evaluated after the behavioral tests. Three rats were excluded due to either HI-derived debilitation and death or sampling failure.

**Figure 9:**
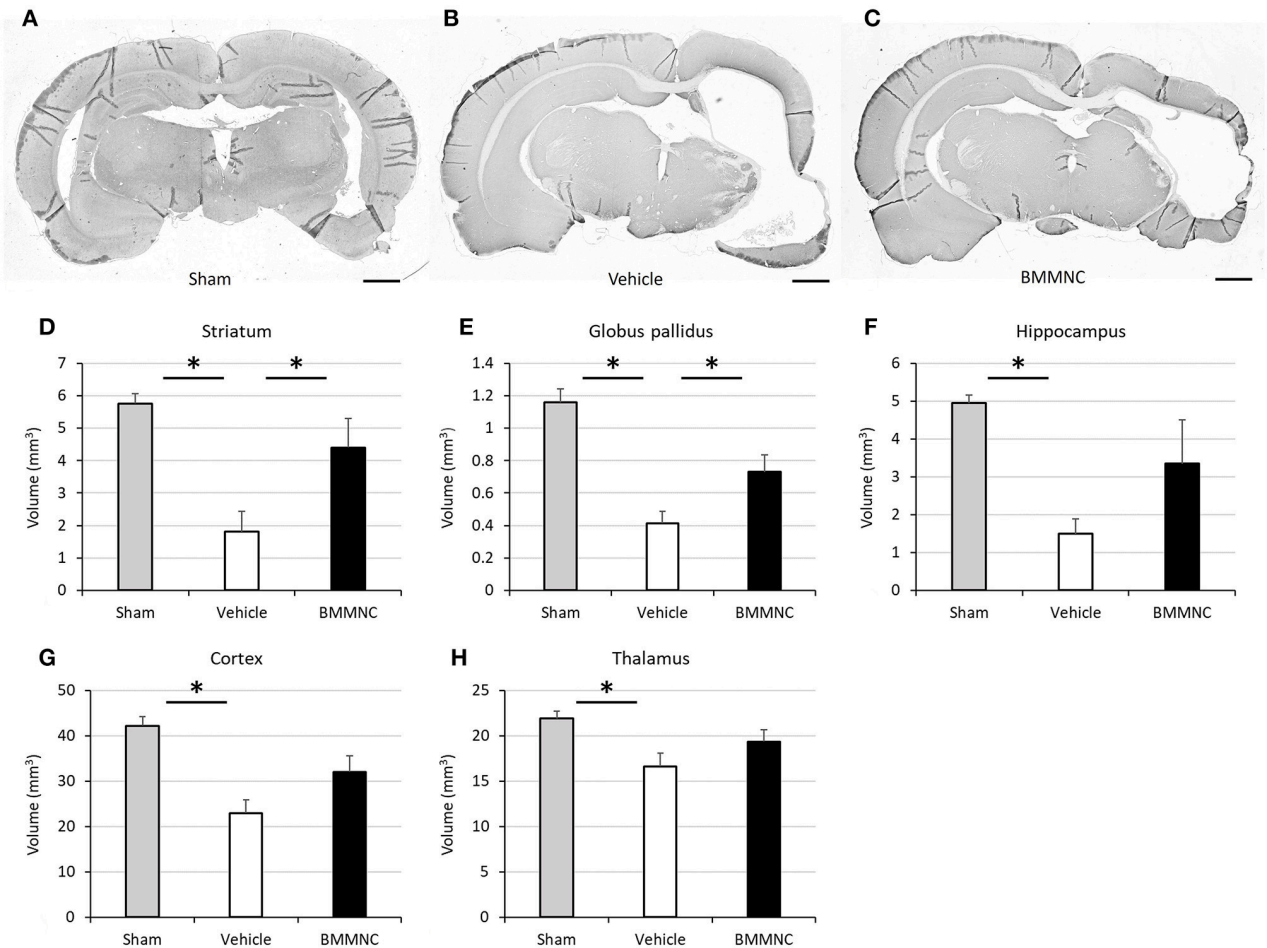
Effect of BMMNC on histological changes after HI. The volumes of ipsilateral striatum and globus pallidus, hippocampus, thalamus, and cortex were evaluated according to the Cavalieri principle. **(A–C)** Representative photomicrographs of the brain stained for MAP2 from sham **(A)**, vehicle- **(B)**, and BMMNC-treated rats **(C)** 6 weeks after HI. Bar = 1,000 μm. The vehicle groups exhibited decreased tissue volume in every region **(D–H)**. The tissue volumes of ipsilateral striatum **(D)** and globus pallidus **(E)** were significantly larger in the BMMNC group than in the vehicle group. No significant difference was found between the BMMNC and vehicle groups regarding the volumes of hippocampus **(F)**, thalamus **(G)**, and cortex **(H)**. Data represent the mean ± standard error of the mean (sham, *n* = 9; vehicle, *n* = 8; BMMNC, *n* = 7) *p < 0.05, Dunnett's test.

Representative photomicrographs stained for MAP2 are shown in Figures 9A–C. Two-way ANOVA showed a statistically significant treatment effect of BMMNC on brain volumes in the affected side after HI (*p* < 0.05). In the analyses of individual regions, the volume of every region was reduced in the vehicle group (Figures 9D–H), and the tissue volumes of ipsilateral striatum and globus pallidus were significantly larger (reduced tissue loss after HI) in the BMMNC group than in the vehicle group (Figures 9D,E). The volumes of hippocampus (Figure 9F), thalamus (Figure 9G), and cortex (Figure 9H) in the affected side appeared to be larger but they were not statistically significant (*p* = 0.11, 0.21, and 0.06, respectively).

## Discussion

In the present study, we demonstrated the safety of intravenous BMMNC administration and its therapeutic effect in reducing the expression of various acute injury biomarkers in the brain using a neonatal HIE rat model. We further showed improvements in the brain infarct volume and limb paralysis.

First, we showed that a single intravenous injection of BMMNC did not cause death to rat pups after HI insult. Cell therapy with BMMNC administration has been gradually developed for several diseases (26–28), although there were few reports regarding the safety of BMMNC intravenous administration to brain-injured rodents during the neonatal period. We previously reported intravenous deliveries of BMMNC with neonatal mice (29). We showed that the BMMNC administrated intravenously was relatively less confined to the lung, which can lead to serious complications, compared with mesenchymal stem cells. In the previous study, we focused on only the acute phase and did not further evaluate the safety. However, the present study demonstrated the safety of BMMNC administration from acute to chronic phase in neonatal rats. BMMNC can be further developed for clinical applications in neonatal HIE. BMMNC administration for cerebral palsy has been evaluated in some clinical trials (30, 31). It appeared safe for human use, but such data were mainly based on patients from early childhood to adolescence but not in the neonatal period. Here, we demonstrated a good survival rate for longer period as well as just after the administration, which supports the safety of BMMNC administration during the neonatal period.

Second, we showed that BMMNC administration attenuated the expression of several acute injury biomarkers. Active caspase-3 is known to play an important role in the apoptosis pathway (32, 33). Once neuronal damage occurs, activated microglia accumulate in the ischemic core and express ED1 (34). Franco et al. (35) has already reported that BMMNC suppresses apoptosis and microglia activation in adult rats. They showed a significant decrease of active caspase-3 positive cells and ED1 positive cells in the ischemic brain after BMMNC treatment. To the best of our knowledge, our study is the first to report anti-apoptotic effects of BMMNC in a neonatal HI rat model. It suggested that BMMNC exhibited therapeutic effects by preventing the acute inflammatory response for newborns as well as for adults.

Finally, we confirmed the therapeutic effects of BMMNC according to histological and motor functional aspects. BMMNC mitigated the loss of brain tissue after HI and reduced the RH/LH print area ratio, reflecting an improvement in gait. Previous studies have reported its beneficial effects on histological and behavioral outcomes using adult rodents (36–38), although few studies reported this effect in newborn rats. Brenneman et al. reported that administration of autologous BMMNC significantly reduced the brain infarct volume in young and middle-aged rats (37). Vahidy et al. (39) conducted a systematic review and meta-analysis and reported that BMMNC had a beneficial effect on histological outcomes in animal ischemic stroke models. Clinical trials have already been performed, but their participants are limited to adults (40). Our study suggested the clinical benefits and application of BMMNC in newborns.

Several publications are currently available on stem cell therapy in the developing brain; Although most publications have reported a positive effect of that treatment (12, 13, 41–43), Dalous et al. (44) have shown that an intraperitoneal injection consisting of human umbilical cord blood mononuclear cells increased the size of the brain lesion in an animal model of excitotoxic brain injury by revealing that the cells increased inflammatory cytokines, which were associated with the aggravation of the lesion. Since the cell administration can, by itself, increase the levels of inflammatory cytokines, it is difficult to interpret how that increase affects the lesion (45). Intravenous infusion of mesenchymal stem cells is known to induce an inflammatory response. We have previously shown that the use of mesenchymal stem cells increased the levels of several inflammatory cytokines, including tumor necrosis factor-α, but still achieved a treatment effect (46). The same study revealed no exacerbation when administration was performed through an intravenous injection. Taken together, these results suggest that the response of cytokines and their effect on the lesion vary with the type of brain injury, the type of stem cells, and/or the administration route.

There are some limitations in the present study. There were no significant differences in some behavioral evaluations, i.e., rotarod treadmill and active avoidance. Iihoshi et al. (18) has reported the improvement of motor function in the treadmill test using a cerebral ischemic adult rat model. Their injected dose (1.0 × 10^7^ cells/rat) was higher than ours even after considering the difference of body weight between adult and neonatal rats. In studies using other types of stem cells with the neonatal HI model, de Paula et al. showed that the treatment effect of the stem cells was dose-dependent (47), and van Velthoven et al. demonstrated that repeated administration exerted a better outcome (48). There is a possibility that our dose and/or times might not be enough to improve behavioral outcomes, including motor and cognitive functions. Some reports have demonstrated the therapeutic effect in the cylinder test, a test for locomotor function (14, 41), which was not performed in the present study. The beneficial effects of BMMNC administration may not appear in motor coordination and cognitive function but be limited to motor function. Further analysis is required to evaluate its mechanism and long-term effect. Another limitation of the present study was the fact that we did not evaluate the synergistic effect of BMMNCs and hypothermia. Since hypothermia is an established, standard therapy for moderate to severe HIE, BMMNCs monotherapy without concomitant hypothermia is unlikely to be performed in clinical practice. Evaluating the combination therapy is therefore necessary and should be addressed by further studies, as well as different types of stem cells (49).

The present study clarified the safety of intravenous BMMNC administration and its therapeutic efficacy in a neonatal HI rat model. Transplantation of autologous stem cells has multiple advantages; ethical issues and possible immune responses associated with allogenic transplantation can be avoided. Unlike UCBCs, BMMNC can be collected even after admitting/transfer to a neonatal intensive care unit. BMMNC might be a good candidate to treat NE. Further studies using different protocols (e.g., various doses, repeated administration, and combination with hypothermia or some other treatments) are needed to elucidate a more detailed mechanism.

## Conclusions

A single intravenous injection of BMMNC 24 h after HI produced morphological and functional improvement. Our findings confirmed that the intravenous injection of BMMNC can be a novel treatment for HI brain injury.

## Author contributions

YoS, TK, AM, YuS, TS, and TH were actively involved in experiments. MY and HH were involved in behavioral experiments. YoS, TK, KN, MT, and MH conceptualized and designed this study. YoS, TK, KN, and MT interpreted the data. TK and KU drafted the initial manuscript, and YoS, AH, KN, MT, and MH critically reviewed the manuscript. AH conducted the statistical analysis. All authors approved the final manuscript as submitted and agree to be accountable for all aspects of the work.

### Conflict of interest statement

The authors declare that the research was conducted in the absence of any commercial or financial relationships that could be construed as a potential conflict of interest. The handling editor is currently co-organizing a Research Topic with one of the authors MT, and confirms the absence of any other collaboration.
